# The diagnostic challenge of pulmonary tumour thrombotic microangiopathy as a presentation for metastatic gastric cancer: a case report and review of the literature

**DOI:** 10.1186/s12885-015-1467-7

**Published:** 2015-06-03

**Authors:** Andrew LK. Ho, Patryk Szulakowsi, Waria HS. Mohamid

**Affiliations:** Lister Hospital, East and North Hertfordshire NHS Trust, Coreys Mill Lane, Stevenage Hertfordshire, SG1 4AB UK

**Keywords:** Pulmonary tumour thrombotic microangiopathy, Gastric adenocarcinoma, Metastatic complications, Progressive dyspnoea

## Abstract

**Background:**

Pulmonary tumour thrombotic microangiopathy (PTTM) is a rare complication of metastatic cancer with a distinct histological appearance which presents with dyspnoea and pulmonary arterial hypertension and leads to death in hours to days. It is a challenging diagnosis to make ante mortem, in part due to the rapid clinical decline. Herein, we report a case of a young woman initially felt to have pulmonary sarcoidosis but who then died eight days later from what was found at post mortem to be PTTM.

**Case presentation:**

A 41 year old Caucasian woman presented with progressive dyspnoea. Computed tomography of her thorax showed diffuse tiny centrilobular nodules in a tree-in-bud appearance along with small volume mediastinal lymphadenopathy. A presumptive diagnosis of pulmonary sarcoidosis was made; bronchoscopy with transbronchial lung biopsy was arranged to confirm the diagnosis. However, she rapidly deteriorated and died eight days later. Post mortem examination revealed metastatic poorly differentiated gastric adenocarcinoma with PTTM being the final cause of death.

**Conclusion:**

This case demonstrates the diagnostic difficulties in such a rare and rapidly fatal oncological complication; a greater awareness amongst clinicians may help make a positive diagnosis in the short window of time available. Little is known about its pathogenesis, and even less about optimal management strategies. We review the literature to demonstrate the clinical characteristics that might provide clues towards an ante mortem diagnosis, and highlight how imatinib may provide the key to treating PTTM.

## Background

Pulmonary tumour thrombotic microangiopathy (PTTM) is a rare complication of cancer with a prevalence of 1.4 % in one retrospective autopsy series of patients who died of cancer [1]. Clinically, it is characterised by dyspnoea and pulmonary arterial hypertension, which almost invariably progresses to right heart strain and cardiorespiratory arrest in hours to days. Pathologically, there are widespread small tumour emboli; it is distinct from conventional tumour emboli in that there is fibrocellular intimal proliferation. The radiological findings on computed tomography are often of diffuse centrilobular nodular opacities, for which the differential diagnosis is wide.

Herein, we report a case of a young woman who presented with dyspnoea and was initially felt to have pulmonary sarcoidosis. However, she rapidly deteriorated and died eight days later. This case report serves to highlight the diagnostic challenges involved in making a timely diagnosis of PTTM, and reviews the possible management options in this rare illness.

### Case presentation

A 41 year old Caucasian woman who was normally very active presented with progressive dyspnoea over the previous five months with an exercise tolerance deteriorating to twenty yards. She was a never-smoker and her past medical history only mentioned spinal fusion surgery in her thirties for congenital spinal scoliosis. Her only medication was Microgynon (a combined oral contraceptive pill). She denied any cough and was otherwise systemically well. A short course of oral steroids from her GP had temporarily improved her symptoms. Clinical examination was non-contributory; in particular, her chest was clear to auscultation and there was no peripherally palpable lymphadenopathy.

**Fig. 1:**
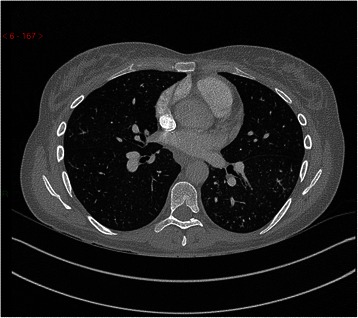
Axial slice of CTPA performed during the patient’s first admission. CTPA demonstrated small centrilobular soft tissue nodules with branching linear opacities forming a tree-in-bud appearance. This is most often caused by obstruction of small airways by, for example, mucus. In PTTM, however, it is caused by tumour cells in distal arterioles and the resultant inflammation that follows. The differential diagnosis for pulmonary nodular infiltrates with mediastinal lymphadenopathy, particularly the small volume adenopathy seen in our patient, is wide-ranging. It includes both malignant causes such as lymphoma as well as benign causes such as tuberculosis, inflammatory nodules, and sarcoidosis

Postero-anterior chest radiograph was unremarkable. Computed tomography pulmonary angiography (CTPA) was performed on suspicion of pulmonary embolism and she was given a single therapeutic dose of dalteparin. This demonstrated adequate opacification of the pulmonary arterial tree with no evidence of filling defect. There were diffuse tiny centrilobular soft tissue nodules forming a tree-in-bud appearance with their adjacent vessels, as well as small volume enlarged lymph nodes in the mediastinum (measuring 11 mm in short axis diameter at the left hilum and adjacent to the aortic arch) (Fig. 1). The combination of lymphadenopathy and nodular infiltrates especially along the fissures and peripherally, in conjunction with the clinical presentation, led to a provisional diagnosis of sarcoidosis, although it was noted at the time that the differential diagnosis for this radiological picture is far wider.

An electrocardiogram showed fixed T wave inversion in leads III, aVF and V1. Transthoracic echocardiography showed a mildly dilated right ventricle (RV) with paradoxical motion of the RV septum indicating RV overload (although the estimated pulmonary artery pressures were not significantly elevated). Spirometry showed a normal FEV1 and FVC with an FEV1/FVC ratio of 73.4 %; lung volumes and transfer factors were normal (table 1). Adjusted calcium was within the normal range; serum angiotensin-converting enzyme levels were requested (later reported as normal at 26 IU/l). She was discharged two days later with an appointment for an outpatient bronchoscopy with transbronchial lung biopsy.

**Table 1:** Pulmonary function tests

Spirometry		
FEV1	2.47 L	(80 % predicted)
FVC	3.37 L	(94 % predicted)
FEV1/FVC	73.44 %	
Transfer factors		
TLCO	70 % predicted	
KCO	85 % predicted	
Body plethysmography		
TLC	5.24 L	(97 % predicted)
VC	3.39 L	(93 % predicted)

Pulmonary function tests performed seven days ante mortem, including lung volumes and transfer factors, were unremarkable

She re-presented three days later feeling generally unwell with worsening dyspnoea, nausea, loss of appetite and diffuse headache. She was now tachycardic (100 bpm), tachypnoeic (24/min) and hypoxic (SpO_2_ 93 % on air). Clinical examination was once again non-contributory. Arterial blood gas analysis showed type 1 respiratory failure (PaO_2_ 8.18 kPa with FiO_2_ 21 %). Electrocardiography showed progression of the right ventricular strain pattern with T wave inversion now in leads III, aVF, and V1–4.

She was noted to have an isolated thrombocytopaenia (93 x10^9^/L); there was no clinical or laboratory evidence of a hypercoagulable state. She was supported with oxygen (up to 4 L/min via nasal cannulae) and also started on co-amoxiclav and clarithromycin as she had a mild neutrophilia (13.17 x10^9^/L with C-reactive protein (CRP) 73 mg/l) to cover for an infective cause for her deterioration. Bronchoscopy with transbronchial lung biopsy, previously intended to be an outpatient investigation, was organised as an inpatient to confirm the diagnosis of pulmonary sarcoidosis. Corticosteroid therapy was not initiated in order to maximise the diagnostic yield of lung biopsy.

Two days after her readmission, whilst awaiting bronchoscopy, she developed persistent tachycardia (100–130 bpm) and tachypnoea (22–30/min) but with normal blood pressure and stable peripheral oxygen saturations on 2–4 L/min oxygen. She was noted to have an episode of unresponsiveness similar to an absence seizure lasting twenty seconds, followed by around thirty seconds of abnormal posturing (flexion but no jerking of limbs) and urinary incontinence. Magnetic resonance imaging of her head was performed to exclude neurosarcoidosis; this was normal. Later the following day, eight days since her first presentation, she suddenly deteriorated over a few minutes and went into cardio-respiratory arrest with pulseless electrical activity (PEA)/asystole. Despite a prolonged resuscitation attempt she passed away.

**Fig. 2:**
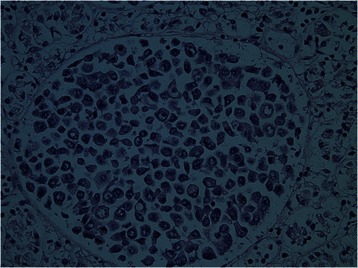
Histological findings of the stomach wall at post mortem. Haematoxylin and eosin (H&E)-stained sections of the anterior gastric wall showed adenocarcinoma with signet ring cell forms

**Fig. 3:**
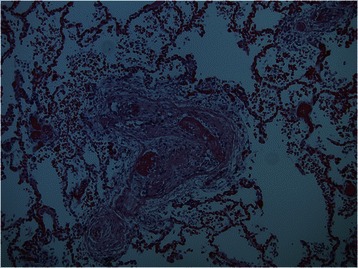
Histological findings of pulmonary vasculature at post mortem. Post mortem examination of the sections taken from both lungs revealed widespread tumour emboli in small and medium sized arterial vessels including sub-segmental branches of the pulmonary arterial tree. This finding was associated with florid intimal fibrocellular proliferation and muscular hyperplasia of the pulmonary vasculature leading to luminal narrowing and stenosis with extensive intra-luminal thrombosis. The pathological conclusion of PTTM is substantiated by the clinical history of the patient's presentation and clinical outcome of the case

Post mortem examination revealed widespread tumour emboli with associated thromboemboli in the subsegmental branches of the pulmonary arterial tree; microscopically, the appearances were consistent with a diagnosis of pulmonary tumour thrombotic microangiopathy. The antecedent cause for this was poorly differentiated adenocarcinoma with signet ring cells in the anterior wall of the stomach (Figs. 2 and 3). Miliary metastases were found in the lung parenchyma as well as metastatic deposits in the kidneys and bone marrow. Sections taken from the brain showed no evidence of occlusive thrombosis or thromboemboli within the intracranial blood vessels; the cause for the neurological event which the patient experienced remains unknown. Retrospective review of the history did not reveal any symptoms indicative of dyspepsia, gastric malignancy, or (other than several weeks of general malaise) occult malignant disease.

## Discussion

PTTM is a rare clinico-pathological entity first described by von Herbay *et al.* in 1990. It is a complication of cancer characterised by widespread microscopic (usually non-occlusive) tumour emboli in the pulmonary arterioles. The distinction from conventional tumour emboli is that it features localised activation of coagulation pathways and the resultant histopathological finding of fibrocellular intimal proliferation with or without secondary thrombosis [2, 3].

For reasons that are not known, the clear majority of cases of PTTM arise from gastric adenocarcinomas, often those of the signet ring cell subtype. In reviewing all cases published in PubMed-indexed journals, we found that gastric cancer accounts for around 60 % of cases, with the remainder comprising lung cancer (9 %), cancer of unknown primary (6 %), breast cancer (3 %), and a few rarely-reported tumour sites.

The clinical presentation is typically of acute dyspnoea. There is a very rapid clinical course, progressing to death in a matter of hours to days. Echocardiography, if performed in a timely fashion, may show features of pulmonary hypertension and right heart strain (with raised pulmonary arterial pressure on right heart catheterisation) [4, 5]. The picture of rapidly progressive dyspnoea often prompts the team managing the patient to organise a CT scan of the thorax. If angiography is performed at the same time, it is usually negative for acute pulmonary embolism [1]. Instead, there are diffuse centrilobular nodular opacities in a tree-in-bud pattern throughout the lung fields, with the possible additional signs of acute cor pulmonale [6–8].

A number of proteins have been implicated as mediators in the development of PTTM. Vascular endothelial growth factor [1, 9] and tissue factor [10] are expressed more frequently in cancer cells leading to PTTM than those with traditional tumour emboli. Platelet-derived growth factor (PDGF) is also expressed more frequently in PTTM-associated cancer cells [9], although the association may be more complex with PDGF overexpression also present in alveolar macrophages and PDGF receptor expression in the proliferating fibromuscular intimal cells [11]. Osteopontin has also been implicated [12, 13]. However, demonstrating overexpression of these factors falls short to construct a model for the pathogenesis of PTTM which remains to be elucidated.

As in our case, PTTM may well occur in patients hitherto unknown to be harbouring malignant disease. The combination of acute dyspnoea as the initial presentation for metastatic disease combined with a rapid deterioration makes ante mortem diagnosis a significant challenge [14]. Indeed, there are only ten case reports in the literature where the diagnosis of PTTM was made whilst the patient was still alive: five were diagnosed by transbronchial lung biopsy [15–19], two by video-assisted thoracoscopic surgery [20, 21], one by CT-guided biopsy [22]; the final two were presumptive ante mortem diagnoses based on proven pulmonary hypertension and the finding of tumour cells from wedged pulmonary artery catheter aspiration [23, 24].

Early ante mortem diagnosis is, however, only the first step towards successful treatment. To date, there are only four case reports in the literature of patients who survived beyond the initial acute phase. The first was in 2007 when a patient with VATS biopsy-proven PTTM from gastric adenocarcinoma was treated with dexamethasone, warfarin, aspirin and fluoropyrimidine-based chemotherapy. The patient's symptoms and radiographic findings resolved, and she was well at six month follow-up [20].

The remaining three cases all experienced significant improvement following the administration of imatinib alongside other medications such as prostacyclin analogs and endothelial receptor antagonists to manage their pulmonary hypertension. These patients had improved pulmonary hypertension but died of either progressive disease [18, 23] or, in one case, influenza infection [24]. There are reports of imatinib being useful in pulmonary arterial hypertension in the non-PTTM setting [25], although its use is currently confined to clinical trials due to the IMPRES study which showed increased morbidity from adverse events despite an improvement in haemodynamics [26]. That imatinib appears to alter the natural history of PTTM lends credence to the theory that PDGF is involved in the pathophysiology. Disappointingly, although PDGF levels fell following imatinib therapy, Ogawa *et al.* did not actually find PDGF expression in either tumour cells or intimal cells. They hypothesised that this was because the biopsy specimen was taken after administration of imatinib and subsequent clinical improvement [18].

An earlier diagnosis (such as on her first hospital admission) is unlikely to have changed the prognosis in this lady with widespread carcinoma, but it should be remembered that definitive diagnosis is not purely an academic pursuit in terminal conditions. There is evidence to suggest that the act of providing information to cancer patients can reduce anxiety [27]. Knowledge of their illness is valuable in that it allows patients to contextualise life decisions [28, 29].

## Conclusions

This case shows the challenges of diagnosing and managing PTTM. This is particularly true for three reasons: the relatively non-specific symptom of dyspnoea; the fact that the patient often does not have a known malignancy; and the rate of clinical decline leading almost inevitably to death. Clinicians should be aware of this as a diagnostic entity, aided by the radiographic findings outlined above as well as the developement of rapidly progressive pulmonary arterial hypertension. More research into the pathogenesis of this condition is clearly required, but given the cases outlined above it seems reasonable that a trial of imatinib should form part of the treatment strategy.

### Consent

Written informed consent was obtained from the next of kin for publication of this case report and any accompanying images. A copy of the written consent is available for review by the Editor of this journal.
