# Erratum to: The diagnostic challenge of pulmonary tumour thrombotic microangiopathy as a presentation for metastatic gastric cancer: A case report and review of the literature

**DOI:** 10.1186/s12885-015-1601-6

**Published:** 2015-08-26

**Authors:** Andrew L K Ho, Patryk Szulakowski, Waria H S Mohamid

**Affiliations:** Lister Hospital, East and North Hertfordshire NHS Trust, Coreys Mill Lane, Stevenage Hertfordshire, SG1 4AB UK

Unfortunately, the original version of this article [1] contained an error. The name of the author Patryk Szulakowski was incorrectly spelt as Patryk Szulakowsi. The correct spelling is Patryk Szulakowski.
